# Rapid-Onset Heparin-Induced Thrombocytopenia Leading to Cardiogenic Shock Due to Left Anterior Descending Artery Embolism During Transcatheter Aortic Valve Replacement

**DOI:** 10.7759/cureus.88622

**Published:** 2025-07-23

**Authors:** Atsuhiro Kitaura, Yukari Yoshino, Hiroatsu Sakamoto, Risa Sakuma, Urara Hirano, Haruyuki Yuasa, Yasufumi Nakajima

**Affiliations:** 1 Anesthesiology, Kindai University Faculty of Medicine, Osaka, JPN; 2 Anesthesiology, Nagoya University, Nagoya, JPN; 3 Anesthesiology and Center for Outcomes Research, University of Texas Health Science Center, Houston, USA

**Keywords:** cardiac shock, cardiothoracic anesthesia, coronary artery embolism, heparin induced thrombocytopenia (hit), middle cerebral artery infarction, transcatheter aortic valve replacement (tavr), va-ecmo

## Abstract

Heparin-induced thrombocytopenia (HIT) is a rare but serious immune-mediated complication of heparin therapy, often resulting in thrombotic events despite adequate anticoagulation. Rapid-onset HIT is a particularly severe variant that occurs within 24 hours of re-exposure to heparin in sensitized individuals with circulating anti-platelet factor 4 (PF4)/heparin antibodies. Although rare, its potential for rapid progression and fatal outcomes necessitates a high index of clinical suspicion, especially in perioperative settings involving routine heparin use. We report the case of an 84-year-old female who underwent transcatheter aortic valve replacement (TAVR) following a prior percutaneous coronary intervention (PCI) with unfractionated heparin exposure two weeks earlier. There was no significant decrease in platelet count before or after the PCI, with values remaining stable from 240,000 to 220,000/μL. Shortly after intraoperative heparin administration during TAVR, the patient developed sudden hemodynamic collapse. Transesophageal echocardiography revealed a transient intracardiac thrombus-like echogenic mass approximately 10 minutes after administration of unfractionated heparin, followed 20 minutes later by acute left ventricular wall motion abnormalities and cardiogenic shock. Emergent venoarterial extracorporeal membrane oxygenation (VA-ECMO) and urgent percutaneous coronary intervention were performed after coronary angiography revealed occlusion of the left anterior descending artery. Subsequently, the TAVR procedure was completed. Surgical complications and ectopic deep vein thrombosis were excluded. The intraoperative 4Ts score ranged from 3 to 4, making the suspicion of HIT equivocal. Although we hesitated to initiate argatroban therapy under these circumstances, heparin was discontinued. Fortunately, the activated clotting time (ACT) remained prolonged. The patient was weaned from VA-ECMO and transferred to the intensive care unit (ICU). Delayed emergence from sedation was noted following ICU admission. A contrast-enhanced computed tomography scan obtained three hours after the conclusion of surgery demonstrated systemic embolization, including infarctions in the right middle cerebral artery and the kidney. The cerebral infarction was deemed ineligible for reperfusion therapy, and a best supportive care approach was adopted. The patient died on postoperative day 10. Based on the patient's clinical course, a final 4Ts score of 6, and a positive immunoassay for HIT antibodies (PF4/heparin enzyme-linked immunosorbent assay (ELISA)), the patient was clinically diagnosed with rapid-onset HIT on postoperative day 4, despite the absence of confirmatory testing using functional assays. Although intraoperative findings suggestive of HIT were observed, distinguishing these from other TAVR-related complications proved clinically challenging, leading to a delayed diagnosis. This case report underscores the importance of recognizing the risk of HIT in patients with a history of heparin exposure and highlights critical areas for improvement in perioperative management strategies.

## Introduction

Heparin-induced thrombocytopenia (HIT) is a serious adverse reaction associated with the use of heparin, a widely prescribed antithrombotic and anticoagulant agent [1]. HIT develops in approximately 0.2-3% of patients exposed to heparin [2,3]. The pathogenesis involves the transient production of IgG-class platelet-activating antibodies that recognize multimolecular complexes formed between heparin and platelet factor 4 (PF4) [4]. This immune-mediated response leads to thrombocytopenia and thromboembolic complications, which, if not properly managed, may result in life-threatening clinical outcomes. It is reported that approximately 10% of patients diagnosed with HIT die during hospitalization [5].

Several subtypes of HIT have been identified [6]. Among these, rapid-onset HIT is characterized by the development of symptoms within minutes to 24 hours following re-exposure to heparin in patients who have been sensitized within the previous 100 days and have circulating HIT antibodies [7]. Rapid-onset HIT accounts for approximately 30% of all HIT cases. This subtype tends to follow a more severe clinical course. Given that heparin is routinely administered in cardiovascular surgery, clinicians must remain vigilant for HIT in such contexts [1].

The diagnosis of HIT, in cases where HIT was suspected, involves initial screening based on clinical features using the 4Ts scoring system, followed by detection of HIT antibodies through immunoassays, and is ultimately confirmed by functional assays [1,8]. While the 4Ts score and the detection of HIT antibodies by immunoassays are useful for screening and supporting the diagnosis, a definitive diagnosis ideally requires functional assays, which can accurately detect pathogenic HIT antibodies [1,8]. However, depending on the timing of symptom onset, differentiating HIT from other perioperative complications can be challenging. This is especially true in structural heart disease interventions such as transcatheter aortic valve replacement (TAVR), where intraoperative hemodynamic instability is common [9], and a variety of procedure-related complications can significantly affect circulation [10]. For instance, intraoperative complications such as coronary artery occlusion or cerebral embolism often occur shortly after heparin administration and may result from surgical factors or embolic events related to TAVR, as well as from thrombotic complications due to HIT. These conditions frequently present with similar clinical manifestations, such as hemodynamic collapse or delayed emergence from anesthesia, making it difficult to distinguish their underlying cause in the acute phase.

In this case report, we present a patient with a history of unfractionated heparin exposure two weeks prior, who developed circulatory collapse within minutes of intraoperative heparin administration during TAVR and could not be resuscitated. Although functional assays were not performed, the diagnosis was considered to be coronary thromboembolism due to rapid-onset HIT, based on the clinical presentation (4Ts score) and the detection of HIT antibodies by immunoassays.

## Case presentation

**Figure 1:**
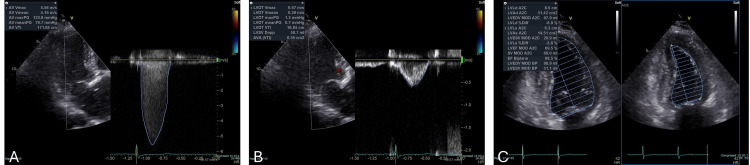
Preoperative transthoracic echocardiography A: Continuous-wave Doppler across the aortic valve showing a peak transvalvular velocity of 5.56 m/s and a peak pressure gradient of 123.8 mmHg. B: Pulsed-wave Doppler in the LVOT, demonstrating an AVA of 0.35 cm² calculated using the continuity equation. C: Parasternal long-axis view indicating a LVEF of 68.5% calculated by the modified Simpson's method. AV: aortic valve, AVA: aortic valve area, LVEF: left ventricular ejection fraction, LVOT: left ventricular outlet tract, aortic valve area, PG: pressure gradient, V: velocity, VTI: velocity time integral

An 84-year-old female patient presented with exertional dyspnea one month prior to scheduled TAVR. Transthoracic echocardiography revealed a severely reduced aortic valve area of 0.35 cm² (calculated by the continuity equation), a peak aortic valve pressure gradient of 112 mmHg, and a mean gradient of 74 mmHg. Minimal aortic regurgitation was observed, and left ventricular ejection fraction was preserved at 68.5% (Figure 1).

Based on these findings, symptomatic severe aortic stenosis was diagnosed. The patient exhibited mild cognitive impairment but was able to walk and perform basic self-care. However, she experienced dyspnea even during short-distance walking on level ground. Considering her age and personal preference, TAVR was planned.

**Figure 2:**
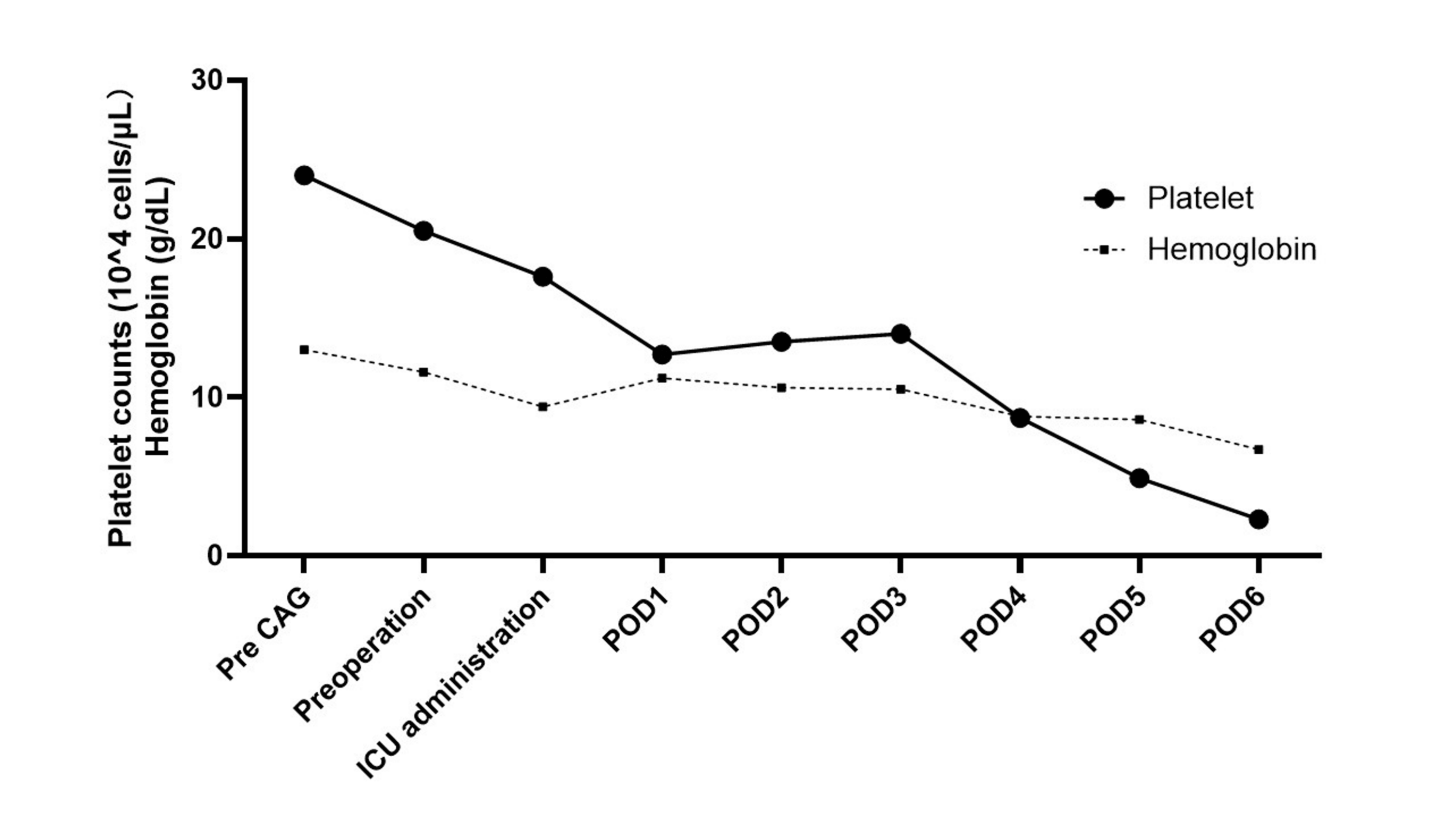
Changes in platelet count A slight decrease in platelet count was observed on the day of surgery; however, this trend paralleled the changes in hemoglobin levels, suggesting the influence of bleeding and hemodilution associated with the induction of extracorporeal membrane oxygenation. On postoperative day 1 (POD1), a marked decrease in platelet count was observed, which appeared to be independent of hemoglobin trends. CAG: coronary angiography, ICU: intensive care units, POD: postoperative day

Two weeks prior to the procedure, preoperative coronary angiography revealed no lesions in the left anterior descending artery (LAD) but showed severe stenosis in the left circumflex artery, for which percutaneous coronary intervention (PCI) was performed. Dual antiplatelet therapy with aspirin and clopidogrel was initiated. Unfractionated heparin was used during PCI, without evidence of thrombocytopenia thereafter (Figure 2).

On the day of surgery, TAVR was performed under general anesthesia. The right femoral artery, noted for favorable vascular anatomy, was selected as the access site. Anesthesia induction was achieved with midazolam 0.06 mg/kg, maintained intraoperatively with 3% desflurane and remifentanil at 0.05 μg/kg/min. During the anesthesia, norepinephrine was also administered concurrently at approximately 0.03 to 0.05 µg/kg/min. Local anesthesia was administered with 10 mL of 1% lidocaine. The right femoral artery was surgically exposed to secure vascular access, and cannulation of the left femoral artery and vein was performed. Heparin was administered at 100 units/kg, resulting in an activated clotting time (ACT) of 388 seconds. Ten minutes after heparin administration, transesophageal echocardiography identified a thrombus-like structure within the left ventricle, which promptly resolved spontaneously (Video 1).

**Video 1:** Transesophageal echocardiographic image of the mid-esophageal left ventricular outflow tract view after heparin administration. A newly appeared, mobile mass suggestive of thrombus is observed in the left atrium.

This finding was reported to the surgical team, and subsequent imaging confirmed no residual or recurrent intracardiac thrombus; the procedure was therefore continued. Twenty minutes following heparin administration, balloon pre-dilation was performed using a 20 mm balloon under rapid pacing (VVI: 180 bpm, pacing duration: 17 seconds). Immediately thereafter, the patient developed shock. Transthoracic echocardiography revealed left ventricular wall motion abnormalities (Video 2).

**Video 2:** Transesophageal echocardiographic image after pre-dilatation balloon aortic valvuloplasty Mid-esophageal left ventricular outflow tract view.  Newly developed significant hypokinesis of the left ventricular wall, particularly involving the interventricular septum and anterior wall, was observed.

Cardiopulmonary resuscitation with chest compressions and adrenaline administration was initiated. Concurrently, veno-arterial extracorporeal membrane oxygenation (VA-ECMO) was established via cannulation of the right femoral artery and left femoral vein. Coronary angiography via the left femoral artery identified occlusion of the LAD (Video 3).

**Video 3:** Left coronary angiography in the right anterior oblique (RAO) view following extracorporeal membrane oxygenation (ECMO) initiation No opacification of the left anterior descending artery (LAD) from its origin was observed.

Urgent PCI was performed, achieving reperfusion and subsequent improvement in wall motion abnormalities. As the ACT was adequately prolonged, no further unfractionated heparin was administered thereafter. After repositioning the ECMO return cannula to the left femoral artery, a self-expanding CoreValve 29 mm prosthesis was implanted via the right femoral artery. ECMO was successfully weaned immediately following TAVR. Vascular injury at the ECMO cannulation site was managed with endovascular angioplasty, concluding the procedure. The patient was transferred to the intensive care unit (ICU) under sedation with dexmedetomidine and 0.3 μg/kg/h intravenous fentanyl via endotracheal intubation.

**Figure 3:**
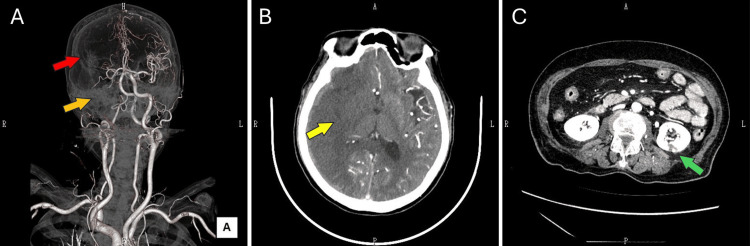
Contrast-enhanced CT image obtained three hours after the ICU administration A: 3D reconstructed CT Angiography image. B: Axial contrast-enhanced CT image of the head at the level of the anterior and posterior horns of the lateral ventricles. C: Axial contrast-enhanced CT image of the abdomen at the level of the kidneys. In A, contrast filling defects are observed in the right internal carotid artery (orange arrow) and the right middle cerebral artery (red arrow). In B, a large area of contrast defect and hypodensity is noted in the right middle cerebral artery territory (yellow arrow). In C, a partial infarction of the left kidney is observed (green arrow).

Despite gradual reduction of sedation to promote awakening, the patient remained unresponsive, exhibiting no voluntary movement of the left side. Contrast-enhanced computed tomography (CT) performed three hours postoperatively revealed occlusion at the origin of the right middle cerebral artery (MCA), extensive perfusion defects within the right MCA territory, parenchymal hypodensity consistent with cerebral infarction, and partial renal infarction (Figure 3).

Neurology consultation concluded the cerebral infarction was established and that reperfusion therapy was not indicated. After family counseling, best supportive care (BSC) was adopted in view of advanced age and poor prognosis. Postoperative tests showed a decreased platelet count of 109,000/μL, which was attributed to bleeding and dilutional effects. In light of the occurrence of multiple embolic events intraoperatively despite adequate anticoagulation, screening for HIT antibodies was performed. On postoperative day (POD) 1, a decrease in platelet count exceeding 30% was observed, followed by a transient recovery the next day. However, by POD 2, cerebral edema had progressed, culminating in brain herniation. From POD 4 onward, recurrent thrombocytopenia was noted, with the platelet count decreasing by more than 50% from the preoperative baseline, although it did not fall below 20,000/μL. Retrospective analysis revealed that the HIT antibody test performed on the day of surgery had returned positive, thereby confirming the diagnosis of HIT. From POD 6, the patient developed diabetes insipidus and gradually showed signs of hemodynamic collapse. The patient died on POD 10.

## Discussion

In the present case, the patient had a history of unfractionated heparin administration two weeks prior to TAVR. Serial preoperative platelet counts demonstrated no significant fluctuations. During the TAVR procedure, transesophageal echocardiography (TEE) detected a transient intracardiac echogenic haze and thrombus formation within 10 minutes of unfractionated heparin administration. Notably, the ACT was adequately prolonged. Twenty minutes following unfractionated heparin administration, embolization to the LAD occurred, precipitating cardiogenic shock. Postoperative evaluation revealed additional embolic events, including infarctions of the right cerebral hemisphere and the right kidney, indicating a pattern of multifocal embolism. Immunologic testing for HIT antibodies performed on the day of surgery was positive. The 4Ts score was 3-4 on the day of surgery, increased to 5 by POD 1, and reached 6 by POD 4, coinciding with marked thrombocytopenia. In Japan, functional assays of HIT antibodies are available only at a limited number of research institutions and are not covered by the national health insurance system. Therefore, considering the patient’s expected prognosis, these tests were not performed. Functional assays for HIT antibodies were not performed in this case. Based on the temporal course and clinical findings, the diagnosis of rapid-onset HIT was established.

Rapid-onset HIT accounts for approximately 30% of all HIT cases [6,7]. It arises when heparin is re-administered to a patient already harboring circulating HIT antibodies. This subtype typically manifests within 24 hours of heparin re-exposure and is known to be associated with more severe clinical outcomes. In our case, this was evidenced by the development of cardiogenic shock and fatal cerebral infarction [7].

The patient’s preoperative history of heparin exposure and clinical context classified her as an intermediate-risk candidate for HIT prior to TAVR, and a high-risk patient postoperatively due to valve replacement surgery [1]. Accordingly, platelet count monitoring was undertaken. Platelet count monitoring is recommended for screening HIT in patients who have received heparin [11-13]. Although immunoassays for detecting HIT antibodies have high sensitivity, their specificity is limited, and false-positive results are common in the absence of thrombocytopenia. Therefore, it is considered appropriate to perform immunoassays only in patients who exhibit a confirmed decrease in platelet count. Moreover, in Japan, functional assays for HIT antibodies are available only at a limited number of specialized research institutions, making them impractical for routine screening. No thrombocytopenia was observed preoperatively; therefore, the use of alternative anticoagulants such as argatroban or bivalirudin was not considered. And although interpretation of platelet counts immediately after surgery was complicated by ECMO support and bleeding-related dilutional effects, no significant platelet drop was observed immediately post-procedure. A platelet count reduction exceeding 30% was first noted on POD 1. Thus, early detection by routine platelet screening, as recommended in the literature, was not achieved. Consequently, the intraoperative embolic events provided the only clinical opportunity for timely diagnosis and intervention.

The cerebral embolism was presumed to have occurred early during TAVR, as supported by CT imaging findings. A hypodense area was observed on CT, indicating that more than six hours had elapsed [14], rendering the patient ineligible for reperfusion therapy [15]. To improve patient outcomes in such cases, rapid diagnosis of HIT and cerebral infarction intraoperatively or immediately postoperatively is essential. However, in this instance, a high index of suspicion for HIT was delayed. Retrospectively, the initial detection of an intracardiac thrombus-like echogenic mass on TEE represented the first critical opportunity to suspect HIT. Temporarily suspending the TAVR procedure at that time to investigate the cause would have been ideal, allowing for earlier initiation of HIT-specific therapy. Unfortunately, the transient and fleeting nature of this echocardiographic finding led to it being underestimated. The highly standardized and rapid nature of the TAVR procedure may have discouraged the surgical team from taking additional time to investigate the cause of the intraoperative events. Furthermore, insufficient awareness of HIT among the surgical team at the time may have contributed to the unfavorable outcome. In response, we revised our institutional protocol to ensure that the possibility of HIT is considered whenever unexpected evidence of new thromboembolic phenomena is encountered.

The next diagnostic opportunity was the initial presentation of an evident embolic event - occlusion of LAD - accompanied by cardiogenic shock. Unfortunately, this event occurred concurrently with balloon pre-dilatation involving rapid pacing. Given that balloon pre-dilatation itself carries an inherent risk of hemodynamic collapse, it was necessary to consider differential diagnoses beyond HIT at the time of circulatory failure. These included more commonly encountered complications such as annular rupture, severe aortic regurgitation, transient myocardial ischemia, and coronary artery occlusion due to causes other than HIT. These factors complicated the immediate clinical diagnosis of HIT based solely on presentation. Coronary obstruction due to device malposition was ruled out as the cause of the coronary occlusion; however, the origin of the embolic material could not be identified. Furthermore, the emergent nature of LAD occlusion necessitated prioritizing life-saving interventions, including establishment of VA-ECMO support, urgent PCI, and vascular repair for ECMO cannulation site injury. This sequence of events delayed HIT diagnosis by approximately three hours. Additionally, the patient’s unstable postoperative condition led to deferred awakening, resulting in a lost opportunity for earlier detection of cerebral infarction and possible reperfusion therapy. Earlier awakening may have facilitated more prompt identification of cerebral embolism and potentially rendered the patient eligible for intervention. It may also have facilitated earlier recognition of multifocal embolism and timely management of HIT. Following this case, a protocol change was implemented to ensure routine consciousness assessment in all patients post-TAVR [16]. Regardless of sedation or general anesthesia, the use of anesthetics that are short-acting and have antagonists present, such as remimazolam [17], may also be useful in the examination of neurologic findings [18].

## Conclusions

Rapid-onset HIT can manifest abruptly with life-threatening complications. In patients with a history of heparin exposure, HIT should remain a consistent differential diagnosis, particularly when thrombotic events occur shortly after re-exposure. Furthermore, when HIT is suspected, heparin should be discontinued promptly, and alternative anticoagulation should be initiated without delay. This case underscores the limitations of relying solely on platelet count monitoring and highlights the critical importance of promptly investigating any clinical signs suggestive of HIT. In the perioperative setting - where general anesthesia and surgical factors often obscure early symptoms - heightened clinical vigilance is essential to ensure timely diagnosis and initiation of appropriate therapy.
